# Effects of a Mobile Health Intervention to Promote HIV Self-testing with MSM in China: A Randomized Controlled Trial

**DOI:** 10.1007/s10461-019-02452-5

**Published:** 2019-03-09

**Authors:** Xiaofang Zhu, Wenhan Zhang, Don Operario, Yue Zhao, Anxia Shi, Zhihua Zhang, Pan Gao, Ashley Perez, Jun Wang, Nickolas Zaller, Cui Yang, Yehuan Sun, Hongbo Zhang

**Affiliations:** 1 School of Public Health, Anhui Medical University, 81 Meishan Road, Hefei, China; 2 Department of Behavioral and Social Sciences, Brown University School of Public Health, 121 South Main Street, Providence, RI 02912 USA; 3 Department of Health Behavior and Health Education, University of Arkansas Fay W. Boozman College of Public Health, Little Rock, AR USA; 4 Department of Health, Behavior and Society, Johns Hopkins Bloomberg School of Public Health, Baltimore, MD USA

**Keywords:** HIV self-testing, Men who have sex with men (MSM), Mobile health (mHealth), China

## Abstract

This study tested a mobile health (mHealth) intervention program entitled *WeTest*, delivered via the WeChat mobile app, to promote oral HIV self-testing (HIVST) among MSM in Hefei, China. A total of 100 MSM participants enrolled, completed baseline assessment, were randomly assigned to intervention or control, and completed 6-month follow-up surveys. Intervention participants (n = 50) received two oral HIVST kits and access to *WeTest*, a private WeChat group which provided app-based messages and referrals to health services related to HIV. Control participants (n = 50) received two oral HIVST kits only. All participants received instructions to upload photographic results of their oral HIVST, which were sent to the project counselor via a secure WeChat online portal; immediate contact and referrals were made to any participants who tested HIV-positive. In GEE analyses adjusting for time effects and baseline confounders, intervention participants had significantly higher rates of HIV testing (adjusted rate ratio RR = 1.99, 95% confidence interval (CI) 1.07–3.84) and, in particular, higher rates of testing via oral HIVST (adjusted RR = 2.17, 95% CI 1.08–4.37) compared with the control group. Significant time effects were also found such that all participants, regardless of group allocation, had significantly higher rates of reporting consistent condom use with main partners (adjusted RR = 18.13, 95% CI 5.19–63.31) and with non-main partners (adjusted RR = 5.33, 95% CI 2.35–12.08). Findings from this study provide evidence for the feasibility, acceptability and preliminary effects of this mHealth approach to promoting oral HIVST among MSM in China.

## Introduction

The WHO recommends that all sexually active men who have sex with men (MSM) should take an HIV test every 6–12 months [1]. HIV testing provides an important opportunity to motivate behavioral risk reduction for MSM who test HIV-negative and is a crucial step in the linkage to treatment and care for MSM who test HIV-positive [2, 3]. Low rates of testing and undiagnosed HIV infection pose substantial risks for furthering the growth of the HIV epidemic [4, 5]. Achievement of the 90–90–90 target (90% detection of all HIV cases, 90% of people living with HIV on ART, 90% of people on ART being virally suppressed) could substantially disrupt HIV transmission at the population level [6]. However, in many parts of the world, suboptimal rates of HIV testing among MSM is a key barrier to this target.

MSM are among the key populations at highest risk for HIV infection in China. MSM in China accounted for 25.5% of the country's newly identified HIV cases and AIDS patients in 2017 [7], and MSM infection rates in China are expected to continue to rise in the absence of effective population-specific prevention programs [8]. Despite a national HIV prevention plan that prioritizes targeted HIV testing services for MSM, HIV testing rates among MSM in China remain low. A systematic review showed that 62% of Chinese MSM had not been tested in the past 12 months and about half of them had never been tested for HIV in their lifetime [9].

Stigma has been identified as a primary barrier to the utilization of HIV prevention and testing services among MSM in China [10–12]. HIV testing rates among MSM are further challenged by limited HIV knowledge, low perceived risk for HIV, low knowledge about HIV testing sites, inconvenient HIV testing clinic times, and fear of receiving a positive HIV diagnosis [12, 13]. Efforts to improve privacy, confidentiality, and more convenient testing services are important to engage MSM in HIV testing and risk reduction [10, 14].

HIV self-testing (HIVST) offers an approach to improve testing rates among MSM in China. Due to features such as its convenience, privacy, and ability to receive prompt results, HIVST may be an effective technology to facilitate HIV testing and identify HIV-infected people at an earlier stage of their disease, especially for people who have never been tested before [15, 16]. A systematic review identified that self-testers can accurately use and interpret the HIV self-test kit and achieve results comparable to those obtained by health-care workers when using HIV rapid diagnostic tests [17]. Oral HIV self-testing kits and finger-prick HIV self-testing kits are available in China. Although both testing techniques are acceptable to potential users in China, research demonstrates greater preference for the former because it is more simple to use (e.g., not requiring a self-collected blood specimen) [18–20].

One of the concerns about HIVST is that, due to the autonomous nature of its use, undergoing HIV testing becomes decoupled from pre-test and post-test counseling, confirmatory testing, and linkage to care [10, 21–23]. Strategies are needed to complement HIVST with timely and appropriate HIV knowledge, guidance about using and interpreting the test kit, behavioral risk reduction information, as well as appropriate referrals.

Mobile health technology (mHealth) has been shown to be a feasible and acceptable approach for HIV prevention and treatment among MSM [24–27], and offers a strategy to communicate essential information to HIVST users. In China, WeChat is a widely used multipurpose mobile application that provides free text/chat messaging, news, gaming, e-commerce and financial services, and other capabilities. Because of the ubiquitous use of this mobile application in China, WeChat offers a potential platform for delivering interactive communication messages to HIVST users.

The aim of this current study was to conduct a pilot randomized controlled trial to test an mHealth intervention entitled *WeTest*, which uses WeChat to deliver app-based information to MSM users regarding the use of and interpretation of HIVST kits, as well as other messages about HIV transmission, risk for other STIs, behavioral risk reduction, and the importance of regular HIV testing. This pilot study examined preliminary effects of the intervention on HIV testing and use of the HIVST kits; as a secondary outcome, effects on condomless sexual behavior were examined.

## Method

This study involved a randomized controlled trial comparing MSM in China who received access to the WeChat platform compared to a control group without WeChat access. The study was conducted from September 2017 to June 2018 in Hefei, the capital city of Anhui Province, China. The protocol was registered on ClinicalTrials.gov (NCT03569462).

### *WeTest* Intervention Development

*WeTest* was developed through a series of preliminary research steps to identify appropriate content and app-based procedures to optimize user engagement with the mHealth intervention. Based on the IMB model of HIV behavior change [28], an initial formative study sought to characterize information domains, motivational factors, and behavioral facilitators to promote HIVST and behavioral risk reduction in the target group. We conducted indepth individual interviews to explore prospective users’ preferences with respect to content of HIVST promotion and HIV prevention messages delivered via WeChat [29]. Based on analysis of qualitative data, an interdisciplinary research team worked in collaboration with staff from a community-based, MSM-focused, HIV prevention organization in Hefei to create a library of messages to provide information, motivation, and behavioral recommendations regarding the need for regular HIV testing and condom use among MSM. Eight MSM were then recruited from community venues to participate in cognitive interviews to review the full library of messages, comment on and critique the clarity, interest level, and relevance of each message. After refining a library of initial messages based on this feedback, a beta-version *WeTest* account was created on WeChat. Sixteen MSM were recruited into a 4-week open pilot of the beta-version *WeTest* program, in which they received access to the *WeTest* account and were asked to review the app-based content and regular text-based and infographic messages delivered via the *WeTest* account. After 4 weeks, participants completed exit interviews to describe their experience using the app, comment on and critique the messages, and provide general recommendations to improve future user engagement. Feedback included comments about the timing, frequency, and tone of messages; suggestions about enhancing user interest by including locally relevant news about HIV and sexually transmitted infections (STIs); links to additional web-based content; and recommendations about incorporating multiple communication modalities such as videos and infographics.

### Participant Recruitment

**Fig. 1:**
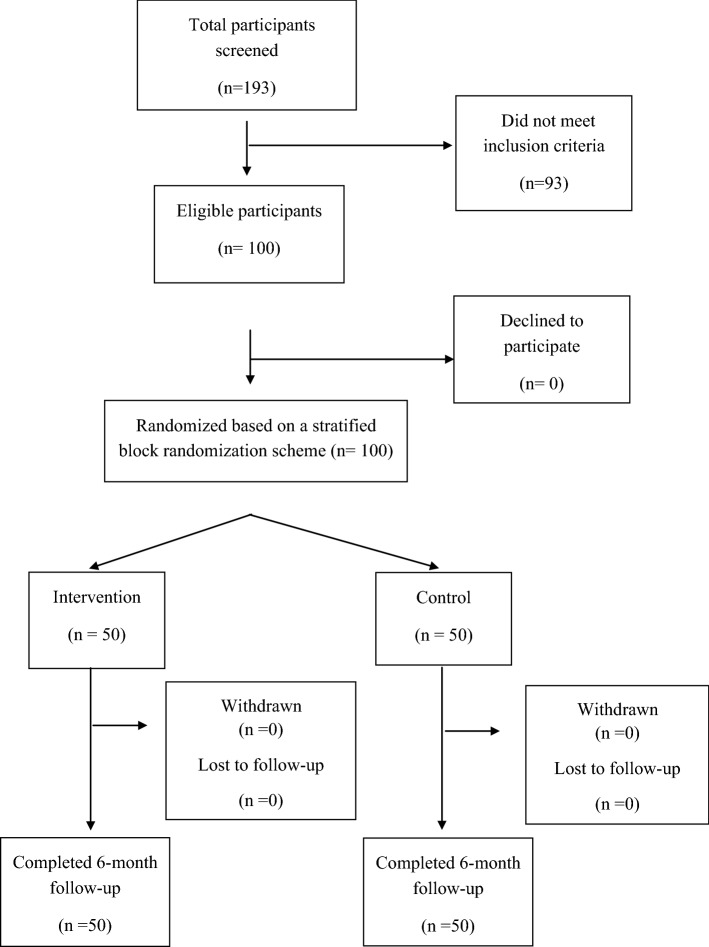
Study flowchart

Participants for the pilot randomized controlled trial were recruited through purposive sampling including outreach at community, commercial and online venues frequented by MSM in Hefei, as well as through community-based referrals through the assistance of a local non-governmental organization serving MSM in Hefei. To be eligible, participants must have met the following inclusion criteria: age 18 or older; Chinese; cis-gender male; a history of unprotected anal sex with another man in the past 6 months; currently residing in Hefei with no intention to leave Hefei during the study period; HIV-negative or status unknown; willing to undergo HIVST; and in possession of a mobile smart-phone with capability to download and use WeChat. Individuals who previously participated in the open pilot were excluded. A total of 193 MSM expressed interest and were screened in person, of which 100 met inclusion criteria. Of the 93 who were excluded, 70 did not have unprotected anal sex in the past 6 months, 2 were younger than 18 years old, 2 had participated in a previous project phase, 3 were unwilling to undergo HIVST, and 8 were not residing in Hefei. In addition, 8 individuals tested HIV positive during baseline procedures (see Procedures), and were immediately referred to a local CDC for confirmatory testing and care and excluded from the study. Figure 1 presents the study flowchart.

### Procedures

After completing informed consent, all participants watched a brief video created by the project team about self-administering the oral HIVST kit and interpreting the test results. A research assistant was initially present in the room to respond to questions arising while watching the video. Following the video demonstration, participants performed the HIVST in private and then completed a 20-minute baseline survey while waiting for their test results. Participants initially read and interpreted their test rests in private, and then the research assistant entered the room to answer questions. In order to maintain a sample of HIV-uninfected participants in the prospective trial, individuals who tested positive at baseline (n = 8) were immediately referred to HIV care and excluded from the study. The research assistant then assigned participants to intervention or control groups, based on an a priori randomization sequence that was managed by the study statistician.

Participants in the intervention group (n = 50) downloaded the *WeTest* mobile app in the presence of the research assistant, who provided an overview of the *WeTest* account features including how to browse messages, videos, and news items on the account. They were instructed to maintain the *WeTest* account for 6 months, during which two new additional messages would be added to the account weekly. *WeTest* messages included brief informational articles about HIV, STIs, and HIV testing; first-person stories about people diagnosed and living with HIV; local data about HIV and STI infections among MSM; news about national policies related to HIV; and stories about general health concerns of MSM. In addition to new content, a video and information text about using the oral HIVST kit were permanently available on the *WeTest* account. The account also included a two-way communication feature in which users could send a text message to a member of the *WeTest* team and receive a reply within 24 h.

Participants in the control group (n = 50) completed the general baseline procedures only—i.e., they watched the video demonstration, self-administered the oral HIVST kit, and completed the baseline questionnaire. Participants in the control condition did not receive access to the *WeTest* account, and did not receive any of the app-based messaging provided to the intervention group.

At the conclusion of the baseline session, all participants were provided two additional oral HIVST kits and received standard information about the need for sexually active HIV-negative or status-unknown MSM to test for HIV every 3 to 6 months. In order to confirm their actual use of the oral test kit and to increase opportunities for two-way communication, the research assistant requested that all participants upload and submit a photograph of the HIVST result via a secure project-specific “customer service account” on WeChat (different from the *WeTest* intervention account); however, participants were informed that this was not mandatory. In the case of an emergency, all participants were informed that a member of the project team was “on call” 24-h via the customer service account to provide immediate response, assistance, and referrals to any participant. In the case of any preliminary HIV positive results, members of the project team were trained in Ministry of Health protocols regarding referrals to the local CDC for confirmatory testing and linkage to care.

Six months following enrollment, participants received a phone call or text message that provided a link to a secure online site for completing the follow-up survey. All participants received 100 RMB (approximately $14 USD) at baseline and at 6-month assessment.

### Measures

#### Socidemographic Characteristics

Participants self-reported their age, education level, employment status, average monthly income in the past 6 months, marital status, current relationship status, sexual orientation, and whether they had *hukou* (official residence registration) in Hefei.

#### HIV Testing

Participants self-reported lifetime and recent 6-month HIV testing behaviors at baseline and follow-up, including use of an oral HIVST kit. As mentioned, participants were asked to upload and submit a photograph of their oral HIVST result, which was included as an additional indicator of testing behavior.

#### Event-Level Sexual Risk Behavior

A secondary outcome was sexual risk behaviors during the past 6 months. Participants described the following sexual behavior characteristics at baseline and at 6-month follow-up: type of relationship with sex partners (main, casual, commercial), partner gender (male, female), and sexual activities (insertive/receptive: oral, anal, vaginal). For each sex act they reported, they reported condom use on a 5-point scale: never, rarely, sometimes, often, always.

### Sample Size Estimation and Statistical Analyses

Previous research reported that about 20% of MSM in China took an HIV self-test in the past 6 months [30]. We anticipated a similar test rate in the control group. A sample size of 38 participants per group was estimated to be sufficient to detect a between-group difference in the HIVST rate of 30% or above, based on 80% statistical power and a two-tailed alpha level of 0.05. In consideration of the potential drop-out rate, we increased the sample size to 50 participants per group, yielding a total sample size of 100.

We first described baseline group differences with respect to participants’ demographic characteristics, HIV testing history, and sexual behaviors. Group differences on categorical variables, such as educational level, occupation and current relationship status, were described using Chi square tests. Group differences on continuous or ordinal variables, such as times of HIV test in the past 6 months, were described using the Wilcoxon rank-sum test. Second, to determine preliminary intervention effects, we conducted multivariable analyses to test our primary hypothesis that participants in the intervention group would engage in higher HIV testing—in particular higher use of HIVST—compared with the control group from baseline to 6-month follow-up. We also explored differences in condom use between the intervention and control groups between baseline and 6-month follow-up. To test our hypotheses, we used generalized estimating equations (GEE) analysis to account for correlations of repeated measures data for both HIV testing and condom use. Relative risk ratios and 95% confidence intervals associated with the “group” variable (intervention vs control) provided the main indicators of preliminary intervention effect sizes for key outcomes. Analyses used an intent-to-treat approach, and adjusted for baseline confounders (group, time, age, education, income, occupation, and hukou). All analyses were conducted in SPSS version 23.0; p-values < 0.05 were considered statistically significant.

## Results

### Descriptive Statistics

A total of 100 participants enrolled (see Table 1 for characteristics reported at baseline). Over half were 18–29 years old (68%), had less than a college degree (53%), earned below 5300 Yuan in the last 6 months (71%), and self-identified as gay or homosexual (78%). There were no statistically significant baseline group differences on the aforementioned characteristics. However, at baseline, control group participants were more likely to report being unemployed students compared with those in the intervention group; hence, multivariable analyses adjusted for occupation.

**Table 1:** Sample Baseline Characteristics

	All (N = 100)	Intervention (n = 50)	Control (n = 50)	*Wald χ* ^*2*^	*P*
	n (%)	n (%)	n (%)		
Age					
18–29	68 (68.0)	34 (68.0)	34 (68.0)	–	1.00
≥ 30	32 (32.0)	16 (32.0)	16 (32.0)		
Education					
Below bachelor	53 (53.0)	28 (56.0)	25 (50.0)	0.36	0.55
Bachelor’s degree and above	47 (47.0)	22 (44.0)	25 (50.0)		
Occupation					
Employed	79 (79.0)	44 (88.0)	35 (70.0)	4.88	0.03
Students	21 (21.0)	6 (12.0)	15 (30.0)		
Incomes (past 6 months)					
5300 Yuan or below	71 (71.0)	33 (66.0)	38 (76.0)	1.21	0.27
5301 or above	29 (29.0)	17 (34.0)	12 (24.0)		
Hukou					
Hefei residents	46 (46.0)	23 (46.0)	23 (46.0)	–	1.00
Non local	54 (54.0)	27 (54.0)	27 (54.0)		
Current relationship states					
Partnered with both male and female	7 (7.0)	5 (10.0)	2 (4.0)	5.38	0.15
Partnered with male only	40 (40.0)	23 (46.0)	17 (34.0)		
Partnered with female only	8 (8.0)	5 (10.0)	3 (6.0)		
Single	45 (45.0)	17 (34.0)	28 (56.0)		
Sexual orientation					
Gay or homosexual	78 (78.0)	41 (82.0)	37 (74.0)	1.04	0.31
Others (bisexual/heterosexual/other)	22 (22.0)	9 (18.0)	13 (26.0)		

A library of 79 messages were created and delivered to participants via WeChat during the intervention period. Based on software diagnostics, approximately 80% of these messages (63 of 79 messages) were read by > 20% of participants and 15% of the messages (12 of 79 messages) were read by > 50% of participants. The most frequently read *WeTest* messages included content concerning MSM diagnosed and living with HIV, drug use, and case studies about HIV infection. Further detailed description about the intervention process, content, and user experience will be provided elsewhere.

During the 6-month follow-up period, 5 intervention group participants “unfollowed” the *WeTest* account and 1 control group participant lost contact with the “customer service account”. These participants discontinued active participation between three to five months following enrollment, and subsequently did not receive *WeTest* messages. Among participants who reported using the oral HIVST kit, 98% (41 of 42 participants) of those in the intervention group and 35% (9 of 26 participants) of those in the control group submitted photographs of their oral HIVST results during the 6-month follow-up period. Two participants received HIV-positive self-test results (both in the control group).

### HIV Testing and Condom Use Behaviors

Table 2 shows descriptive data on self-reported HIV testing behaviors at baseline and follow-up. At baseline, HIV testing history and attitudes towards oral self-testing in the intervention and control groups were statistically equivalent. Most participants at baseline had taken an HIV test of any type in the 6 months (62% intervention, 58% control), but few participants had ever self-administered the oral HIV self-test kit (12% intervention, 22% control). Most participants reported limited trust in results of the oral HIV self-test kit (58% intervention, 68% control) at baseline. Almost all participants reported inconsistent condom use for receptive and insertive anal sex with both main and casual/commercial male sex partners at baseline (see Table 2).

**Table 2:** Past 6-month HIV testing behaviors and condom use behaviors

	Baseline				Follow-up			
	Intervention (n = 50) n (%)	Control (n = 50) n (%)	*Waldχ* ^*2*^ */Z*	*P*	Intervention (n = 50) n (%)	Control (n = 50) n (%)	*Waldχ* ^*2*^ */Z*	*P*
Any HIV Test								
Yes	31 (62.0)	29 (58.0)	0.17	0.68	46 (92.0)	34 (68.0)	9.00	< 0.01
No	19 (38.0)	21 (42.0)			4 (8.0)	16 (32.0)		
Any Oral HIV self-test								
Yes	6 (12.0)	11 (22.0)	1.78	0.18	42 (84.0)	26 (52.0)	11.77	< 0.01
No	44 (88.0)	39 (78.0)			8 (16.0)	24 (48.0)		
Sent photo oral HIV self-test								
Yes	–	–	–	–	41 (82.0)	9 (18.0)	40.97	< 0.01
No	–	–			9 (18.0)	41 (82.0)		
Times of HIV test received								
0	19 (38.0)	21 (42.0)	− 0.35	0.73	4 (8.0)	16 (32.0)	− 2.91	< 0.01
1	13 (26.0)	12 (24.0)			14 (28.0)	14 (28.0)		
2 or more	18 (36.0)	17 (34.0)			32 (64.0)	20 (40.0)		
Trust degree in oral self-test result								
Completely trust	21 (42.0)	16 (32.0)	1.07	0.30	30 (71.4)	11 (42.3)	5.69	0.02
Not completely trust	29 (58.0)	34 (68.0)			12 (28.6)	15 (57.7)		
Condom use with main male sex partner*								
Every time	2 (4.2)	2 (4.6)	0.01	0.91	15 (40.5)	18 (40.0)	0.01	0.96
Not every time	46 (95.8)	41 (95.4)			22 (59.5)	27 (60.0)		
Condom use with casual or commercial male sex partner*	37	38			25	31		
Every time	5 (13.5)	9 (23.1)	1.28	0.26	14 (56.0)	16 (51.6)	0.11	0.74
Not every time	32 (86.5)	29 (76.9)			11 (44.0)	15 (48.4)		
Condom use during receptive anal sex with male sex partner*								
Every time	2 (6.5)	6 (16.2)	1.55	0.21	9 (39.1)	15 (51.7)	0.82	0.37
Not every time	29 (95.5)	31 (83.8)			14 (60.9)	14 (48.3)		
Condom use during insertive anal sex with male sex partner*								
Every time	1 (2.7)	5 (16.1)	3.78	0.05	14 (48.3)	15 (48.4)	0.00	0.99
Not every time	36 (97.3)	26 (83.9)			15 (51.7)	16 (51.6)		

Age, education, incomes, occupation, and hukou were controlled in all GEE models

*Categories do not sum to n = 50 due to missing values

At follow-up, participants in the intervention group compared with those in the control group reported significantly higher prevalence of taking any type of HIV test during the past 6 months (92% vs 68%, *p *< 0.01) and self-administering the oral HIVST kit (84% vs 52%, *p *< 0.01). Intervention participants had a higher prevalence of submitting a photograph of their oral HIVST result compared with control group participants (82% vs 18%, *p *< 0.01), had a higher prevalence of taking an HIV test 2 or more times during the past 6 months (64% vs 40%, p < 0.01), and reported higher trust in the oral HIVST results (71% vs 42%, *p *= 0.02). There were no significant differences between intervention and control groups in self-reported consistent condom use at follow-up, whether with main male sex partners (40% vs 40%, *p *= 0.96) or with casual or commercial male sex partners (56% vs 52%, *p *= 0.74), or during receptive anal sex (39% vs 51%, *p *= 0.37) or insertive anal sex (48% vs 48%, *p *= 0.99).

### Intervention Effects on HIV Testing and Condom Use Behaviors

Table 3 presents GEE models presenting intervention effect sizes with regard to the HIV testing and condom use outcomes. Group, time, age, education, incomes, occupation, and hukou were included in all GEE models. With respect to the primary outcomes, there was a significant increase in HIV testing behaviors (adjusted RR = 1.99, 95% CI 1.03–3.84, p < 0.05), use of oral HIVST (adjusted RR = 2.17, 95% CI 1.08–4.37, *p *< 0.05), and trust in the result of the oral HIVST (adjusted RR = 2.28, 95% CI 1.15–4.51, *p *= 0.02) in the intervention group compared to control. With respect to condom use behaviors, there were no significant differences between intervention and control groups. However, there were significant time effects for all condom use behaviors, such that participants reported more consistent condom use with a main male sex partner (adjusted RR = 18.13, 95% CI 5.19–63.31, *p *< 0.01), with a casual or commercial sex partner (adjusted RR = 5.33, 95% CI 2.35–12.08, *p *< 0.01), during receptive anal sex with any male partner (adjusted RR = 8.03, 95% CI 3.29–19.62, *p *< 0.01), and during insertive anal sex with any male partner (adjusted RR = 9.80, 95% CI 3.78–25.41, *p *< 0.01).

**Table 3:** Effect of intervention on HIV testing and condom use behaviors in past 6 months using generalized estimating equations

	RR	SE	95% Wald *CI*		*Wald χ* ^*2*^	*P*
			Lower	Upper		
HIV test, last 6 mos						
Group (intervention vs control)	1.99	1.40	1.03	3.84	4.18	0.04
Time (follow up vs baseline)	2.96	1.38	1.57	5.58	11.21	< 0.01
Oral HIV self-test, last 6 mos.						
Group (intervention vs control)	2.17	1.43	1.08	4.37	4.77	0.03
Time (follow up vs baseline)	12.43	1.46	5.96	25.93	45.14	< 0.01
Trust degree in oral self-test result*						
Group (intervention vs control)	2.28	1.42	1.15	4.51	5.62	0.02
Time (follow up vs baseline)	2.39	1.40	1.23	4.63	6.63	0.01
Consistent condom use with main male sex partner, last 6 mos						
Group (intervention vs control)	0.90	1.52	0.39	2.06	0.06	0.80
Time (follow up vs baseline)	18.13	1.89	5.19	63.31	20.63	< 0.01
Consistent condom use with casual or commercial male sex partner, last 6 mos.						
Group (intervention vs control)	0.95	1.52	0.41	2.17	0.02	0.89
Time (follow up vs baseline)	5.33	1.52	2.35	12.08	16.06	< 0.01
Consistent condom use during receptive anal sex with male sex partner, last 6 mos^†^						
Group (intervention vs control)	0.40	1.84	0.12	1.32	2.27	0.13
Time (follow up vs baseline)	8.03	1.58	3.29	19.62	20.90	< 0.01
Consistent condom use during insertive anal sex with male sex partner, last 6 mos^†^						
Group (intervention vs control)	0.60	1.62	0.23	1.54	1.17	0.29
Time (follow up vs baseline)	9.80	1.62	3.78	25.41	22.38	<0.01

*Categories: Strongly trust versus do not strongly trust (reference)

^†^Categories: Always versus not always (reference)

## Discussion

This study explored the initial feasibility and effects of a mHealth intervention to promote HIV testing and, in particular, use of HIVST among MSM in China. Overall, results showed that the intervention increased HIV testing behavior, self-administration of HIVST, and trust in results of HIVST results over 6 months of follow-up compared to the control group, but had limited effects on increasing consistent condom use in the intervention group compared to the control group. These findings offer a promising indication that the *WeTest* intervention facilitated use of and trust in oral HIVST as a means for increasing awareness of one’s HIV status.

Preliminary intervention findings are useful in the context of growing acceptance of HIV self-testing among MSM in China. A cross-sectional study of 5996 MSM in Beijing reported that 40% had previously used HIVST kits and 92% were willing to use HIVST kits in the future [30]. Another study involving 2383 MSM found that 16% of MSM in China who engaged in HIVST sought follow-up HIV prevention, testing, counseling, or care services provided at a non-governmental organization or CDC, suggesting that HIVST can offer an important step in improving use of in-person HIV services [21]. Thus, HIVST is a potential platform to achieving China’s 90–90–90 goals, by improving engagement in HIV care services among HIV-infected populations, as well as China’s overall HIV testing and prevention goals.

Findings from this pilot intervention provide preliminary evidence about the feasibility, acceptability, and preliminary effects of mHealth interventions for HIV-related outcomes in China. For example, WeChat has been shown to be a promising mode of delivering messages to promote medication adherence [31] as well as mental health and quality of life [32] among people living with HIV in China. Previous research has described the potential for mHealth interventions to improve engagement in HIV prevention and sexual health risk reduction programs for MSM in China [27]. Nearly all (98%) of those who reported using oral self-test kits in the intervention group uploaded and submitted photographs with their test results to a remote *WeTest* staff counselor via the secure portal. This high submission rate is indicative of the potential utility for remote health workers to counsel, refer, and provide linkages to care for people who test positive. In consideration of the barriers to in-person HIV testing and prevention counseling services described by MSM previously, HIVST coupled with mHealth interventions such as *WeTest* can facilitate comfort performing HIV testing in private along with providing prompt access to referrals to follow-up services and HIV risk reduction messages delivered by a remote counselor.

Notably, participants who enrolled in the study had high rates of recent HIV testing at enrollment, relative to estimates of HIV testing behaviors reported in other studies of MSM in China [9, 12], though fewer had used the HIVST kit. Previous studies have described some of the barriers to using oral HIVST among MSM in China, including low knowledge about oral HIVST as a method for HIV testing, mistrust and doubts about the accuracy of oral self-testing, and poor access to oral HIVST kits [13, 33]. Indeed, one of the broad barriers to use of HIVST is distrust of the HIVST products that are commonly available in China. Anecdotal reports have indicated the availability of oral HIVST kits through online markets in China, and MSM informants have expressed concern about the accuracy and authenticity of HIVST kits purchased through online sources [29]. The higher levels of trust toward oral HIVST results observed in the study may be attributed to our WeChat messages which provided information about the high quality of oral HIVST kits when provided through a reliable source such as CDC-endorsed NGOs or research universities in China. Therefore, efforts to improve access to safe, reliable, accurate and affordable oral HIVST kits in China are essential to achieve the promise of these kits to increase HIV testing behaviors.

The *WeTest* pilot intervention did not achieve higher rates of consistent condom use among intervention participants compared to the control group at follow-up. Self-reported consistent condom use was extremely low in both intervention and control groups at baseline, for each type of sexual behavior assessed. For example, less than 5% reported consistent condom use with main male sex partners, and approximately 20% reported consistent condom use with casual or commercial sex partners. Rates of consistent condom use increased considerably in both groups at follow-up, with approximately half of all participants reporting consistent condom use with main male sex partners and with casual or commercial sex partners, respectively. The higher rates of consistent condom use might reflect the positive effects of self-administering the oral HIVST kit and receiving preliminary HIV test result for all participants at baseline, which may have potentially led participants to reflect of their HIV risk factors and attempt to minimize risk through consistent condom use. However, the higher rates of consistent condom use reported at follow-up might also reflect participant self-report bias and the desire to appear compliant with HIV prevention recommendations.

### Limitations

There are several limitations this study. First, this study used a non-representative sample recruited through targeted physical and online venues. A noteworthy limitation of the sample was that the majority of participants had engaged in HIV testing during the 6 months prior to enrollment, which is not representative of the general MSM population in China. Future research with diversified recruitment methods to reach more high-risk subgroups of MSM samples are needed to determine the generalizable effects of mHealth approaches to promote oral HIVST in China. Second, one of the inclusion criteria stipulated that individuals must agree to self-administer the oral HIVST kit at baseline, which introduced a selection bias against those who were unwilling to do so. This specific criterion was based on our initial concern about the potential misuse or incorrect interpretation among first-time users of the oral HIVST kit, which was a relatively novel device in China and which necessitated an in-person demonstration of the HIVST kit at enrollment to ensure appropriate use. Given our understanding of the relative ease of using the oral HIVST kit, future research might not require selecting participants based on their willingness to self-administer the oral HIVST kit at enrollment. Third, participants received oral HIVST kits free of charge directly through the project, which does not reflect the actual economic and physical barriers to accessing HIVST kits in China [34]; this design feature may have led to an overestimate in the usage of oral HIVST kits among MSM with limited financial resources. Further research examining the cost effectiveness of HIVST can inform the utility of incorporating HIVST at free or discounted prices into the national VCT policy. Fourth, despite the generally high HIVST acceptance level and retention rate of MSM participants observed in the study, there were some participants who discontinued using the app. Privacy was among the noted concerns about the *WeTest* app, and further research is needed to understand how to improve user engagement and retention in the intervention. Although most HIVST users in the intervention group submitted their photographic testing results via the online portal, fewer of the participants in the control group did so. Further research is warranted to understand users’ concerns related to sending HIVST result through electronic portals—via photographs or text—in order to facilitate timely and appropriate responses (e.g., counseling, referrals, linkage to care) at the point of home-based testing. Fifth, this pilot study assessed a small sample size, included short-term self-report outcomes, and lacked biological confirmation of sexual risk reduction outcomes. These methodological design characteristics were likely to have contributed to large confidence intervals. Future studies incorporating rigorous methodological design features such as long-term follow-up, biological outcomes, and a larger sample size are needed to provide stronger evidence of intervention effects. Finally, due to the preliminary nature of this pilot study, we were unable to test mediating variables that are necessary to understand the mechanisms of action that determine behavior change.

## Conclusions

Findings from this study provide preliminary evidence about the feasibility, acceptability, and initial effects of using an mHealth approach to facilitate use of oral HIVST kits among MSM in China. Intervention effects on condom use were not observed; instead, participants in both intervention and control groups reported increased condom use. Future research is needed to test the effects of this mHealth strategy on HIV testing and sexual risk reduction, and will benefit from more representative MSM samples, a longer follow up period, biological or other objective indicators of intervention effects, assessment of theoretical mechanisms, and additional efforts to ensure users’ sense of privacy and safety. Cost-effectiveness analysis can inform policy decisions regarding increasing access to HIVST and incorporating this testing modality in China’s HIV prevention and testing policies. Future HIVST social marketing initiatives in China should also include efforts to address low HIV knowledge, awareness and testing of other highly prevalent STIs, and the role of stigma as a barrier to HIV/STI testing, access and linkage to care and treatment for people who test positive for HIV or STIs, and adherence to treatment for MSM in China.

## Abbreviations

mHealth: Mobile health
MSM: Men who have sex with men
CI: Confidence interval
RR: Rate ratio
HIVST: HIV self-testing
STI: Sexually transmitted infection

## References

[1] World Health Organization (WHO), UNAIDS. Guidance on provider-initiated HIV testing and counselling in health facilities. Switzerland. 2017.

[2] Phillips AN, Cambiano V, Miners A (2015). Potential impact on HIV incidence of higher HIV testing rates and earlier antiretroviral therapy initiation in MSM. AIDS..

[3] Liu Y, Qian HZ, Ruan Y (2016). Frequent HIV testing: impact on HIV risk among Chinese men who have sex with men. J Acquir Immune Defic Syndr.

[4] Kozak M (2013). Late diagnosis, delayed presentation and late presentation in HIV: proposed definitions, methodological considerations and health implications. Antiviral Therapy..

[5] Luo S, Han L, Lu H (2015). Evaluating the impact of test-and-treat on the HIV epidemic among MSM in China using a mathematical model. PLoS ONE.

[6] UNAIDS. 90–90–90 An ambitious treatment target to help end the AIDS epidemic. Geneva Switzerland Unaids Joint United Nations Programme on HIV/AIDS Oct, 2014.

[7] NCAIDS, NCSTD, China CDC (2018). Update on the AIDS/STD Epidemic in China in December, 2017. Chin J AIDS STD. 2018; 24(02):111.

[8] Ge L, Li D, Li P, Guo W, Cui Y (2017). Population specific sentinel surveillance for HIV infection, syphilis and HCV infection in China, during 2010–2015. Dis Surveill..

[9] Zou H, Hu N, Xin Q, Beck J (2012). HIV testing among men who have sex with men in China: a systematic review and meta-analysis. AIDS Behav..

[10] Johnson CC, Kennedy C, Fonner V (2017). Examining the effects of HIV self-testing compared to standard HIV testing services: a systematic review and meta-analysis. J Int AIDS Soc..

[11] Wei C, Cheung DH, Yan H (2016). The impact of homophobia and HIV stigma on HIV testing uptake among chinese men who have sex with men: a mediation analysis. J Acquir Immune Defic Syndr.

[12] Liu Y, Sun X, Qian HZ (2015). Qualitative assessment of barriers and facilitators of access to HIV testing among men who have sex with men in China. AIDS Patient Care STDs..

[13] Tang S, Tang W, Meyers K, Chan P, Chen Z, Tucker JD (2016). HIV and syphilis among men who have sex with men and transgender individuals in China: a scoping review. BMC Infect Dis.

[14] Pyun T, Santos GM, Arreola S (2014). Internalized homophobia and reduced HIV testing among men who have sex with men in China. Asia Pac J Public Health.

[15] Zhang C, Li X, Brecht ML, Koniak-Griffin D (2017). Can self-testing increase HIV testing among men who have sex with men: a systematic review and meta-analysis. PLoS ONE.

[16] Qin Y, Tang W, Nowacki A (2017). Benefits and potential harms of human immunodeficiency virus self-testing among men who have sex with men in China: an implementation perspective. Sex Transm Dis.

[17] Figueroa C, Johnson C, Ford N (2018). Reliability of HIV rapid diagnostic tests for self-testing compared with testing by health-care workers: a systematic review and meta-analysis. Lancet HIV..

[18] Figueroa C, Johnson C, Verster A, Baggaley R (2015). Attitudes and acceptability on HIV self-testing among key populations: a literature review. AIDS Behav..

[19] Marley G, Kang D, Wilson EC (2014). Introducing rapid oral–fluid HIV testing among high risk populations in Shandong, China: feasibility and challenges. BMC Public Health..

[20] Wang X, Wu Z, Tang Z, Nong X, Li Y (2018). Acceptability of HIV testing using oral quick self-testing kit in men who have sex with men. Chin J Epidemiol..

[21] Ren X, Wu Z, Mi G (2017). HIV care-seeking behaviour after HIV self-testing among men who have sex with men in Beijing, China: a cross-sectional study. Infect Dis Poverty..

[22] Stevens DR, Vrana CJ, Dlin RE, Korte JE (2018). A global review of HIV Self-testing: themes and implications. AIDS Behav..

[23] Wood BR, Ballenger C, Stekler JD. Arguments for and against HIV self-testing. HIV/AIDS Research & Palliative Care. 2014, 2014(default):117. 10.2147/HIV.S49083 PMC4126574 25114592

[24] Mulawa MI, Legrand S, Hightow-Weidman LB. eHealth to enhance treatment adherence among youth living with HIV. Current Hiv/aids Reports. 2018;1–14. 10.1007/s11904-018-0407-y PMC6086132 29959649

[25] Schnall R, Travers J, Rojas M, Carballo-Diéguez A (2014). eHealth interventions for HIV prevention in high-risk men who have sex with men: a systematic review. J Med Internet Res.

[26] Devi BR, Syed-Abdul S, Kumar A (2015). mHealth: an updated systematic review with a focus on HIV/AIDS and tuberculosis long term management using mobile phones. Comput Methods Programs Biomed.

[27] Muessig KE, Bien CH, Wei C (2015). A mixed-methods study on the acceptability of using eHealth for HIV prevention and sexual health care among men who have sex with men in China. J Med Internet Res..

[28] Fisher JD, Fisher WA (1992). Changing AIDS-risk behavior. Psychol Bull.

[29] Zhao Y, Zhu X, Perez AE (2018). MHealth approach to promote Oral HIV self-testing among men who have sex with men in China: a qualitative description. BMC Public Health..

[30] Ren X, Wu Z, Mi G, Mcgoogan J, Rou KM, Zhao Y (2017). Uptake of HIV self-testing among men who have sex with men in Beijing, China: a cross-sectional study. Biomed Environ Sci.

[31] Guo Y, Xu Z, Qiao J (2018). Development and feasibility testing of an mHealth (text message and WeChat) Intervention to improve the medication adherence and quality of life of people living with HIV in China: pilot randomized controlled trial. JMIR mHealth uHealth..

[32] Guo Y, Hong YA, Qiao J (2018). Run4Love, a mHealth (WeChat-based) intervention to improve mental health of people living with HIV: a randomized controlled trial protocol. BMC Public Health..

[33] Xu Y, Zhang Z, Li D (2013). Willingness to use the oral fluid HIV rapid test among men who have sex with men in Beijing. China. PLOS ONE..

[34] World Health Organization (WHO). Guidelines on HIV self-testing and partner notification: supplement to consolidated guidelines on HIV testing services. Geneva Switzerland Who Dec, 2016. 27977094

